# Opportunities and risks involved in using ChatGPT to create first grade science lesson plans

**DOI:** 10.1371/journal.pone.0305337

**Published:** 2024-06-17

**Authors:** Wardell Powell, Steven Courchesne

**Affiliations:** 1 Education Department, Framingham State University, Framingham, MA, United States of America; 2 ITS-Education Technology Office, Framingham State University, Framingham, MA, United States of America; Far Eastern University - Manila, PHILIPPINES

## Abstract

Generative AI can potentially support teachers in lesson planning by making the process of generating an outline more efficient. This qualitative study employed an exploratory case study design to examine a specific lesson design activity involving a series of prompts and responses from ChatGPT. The desired science lesson on heredity was aimed at first grade students. We analyzed the process’s efficiency, finding that within 30 minutes we could generate and substantially refine a lesson plan that accurately aligned with the desired curriculum framework and the 5E model of instruction. However, the iterations of the lesson plan included questionable components, missing details, and a fake resource. We discussed the implications of these findings for faculty looking to train pre-service teachers to appropriately use generative AI in lesson planning.

## Introduction

Lesson planning is an essential and time-consuming part of teaching. A lesson plan is a roadmap for the learning process because the plan sets goals for the learning task and aligns these goals with the specific content and assessments that will help students to achieving their learning goals. Preparing a well-designed lesson can be time consuming. Teachers reportedly spend an average of seven hours per week on lesson planning, making it an even more time-intensive task than grading [1]. Generally, teachers can benefit from time saving measures. On average, teachers in the United States reported working 53 hours per week, which includes planning, teaching and grading [2]. Therefore, it is valuable to identify ways to make lesson planning more efficient, without sacrificing quality. To that end, we explored how generative AI can be used as a tool to support teachers in lesson planning.

Generative AI is defined as a large language model, trained on vast amounts of data and capable of generating new content [3, 4]. With its capacity to generate information when prompted, generative AI is a tool that can potentially be used to help teachers effectively plan lessons while saving time. An important feature is a user interface that allows individuals to input prompts and receive responses from the system in natural human language [4]. Although artificial neural networks have been under development since the 1950s [5], the advent of ChatGPT 3.5 in 2022, and the subsequent wave of similar tools such as Google Bard and Microsoft CoPilot [6], facilitated the widespread adoption of generative AI [4].

### Using generative AI in education settings

Researchers have found that generative AI is useful for teachers, to generate course materials and assessments, give suggestions, translate information into other languages, and facilitate assessing student work [7]. With regards to lesson planning, researchers have successfully tested the capacity of ChatGPT to generate lesson plans and ancillary resources, finding that generative AI can serve as a valuable starting point for middle school lessons, including an ESL lesson [8] and a unit comparing renewable and non-renewable energy sources [9].

One approach used to study generative AI in lesson planning was for researchers to prompt ChatGPT to generate hypothetical lesson plans and then conduct document analysis of the outputs [8, 9]. van den Berg and du Plessis prompted ChatGPT to create a plan for a lesson focused on prepositions for sixth grade English language learners [8]. In addition, ChatGPT created a student worksheet and a visual presentation of the generated lesson. The document analysis considered how AI tools can support brainstorming, teachers’ critical thinking about outputs, and exploration of different approaches to their lessons. van den Berg and du Plessis noted that it is important for teachers to critically examine outputs by generative AI, such as to evaluate accuracy, lesson creativity, and context [8]. The generated lesson left certain details to the teacher, such as what images could be appropriate for a warm-up activity and how to make the lesson culturally relevant. The authors argued that using generative AI should be a "starting point, not a final product,” (p. 10) and so teachers should analyze and adapt generated lessons.

A similar study explored how ChatGPT could influence science education, particularly to help teachers with lesson planning [9]. The author employed an exploratory self-study methodology, which involved prompting the generative AI tool and then reflecting on the outputs. Before prompting the generation of the lesson plan, Cooper asked ChatGPT about key characteristics related to effective science teaching, inquired about the relative value of traditional lecturing versus student-centered teaching approaches, and asked whether a teacher should be concerned if a student is failing science [9]. In addition, the author asked ChatGPT to design a teaching unit based on the 5E Model, which involves the following stages of learning: engage, explore, explain, elaborate, and evaluate. The lesson focused on differentiating renewable and non-renewable sources of energy at a middle school level. To support the unit, ChatGPT also produced a rubric and quiz. In response to the philosophical and pedagogical questions about effective science teaching and the importance of supporting struggling students, Cooper determined that ChatGPT aligned with research-informed best practices [9]. ChatGPT also confirmed the importance of caring about failing students and provided helpful suggestions for supporting students. ChatGPT successfully developed a teaching unit based on the 5E model, though Cooper judged the output to be slightly generic because the lesson did not account for the contextual factors, such as student needs and school profile.

### Concerns regarding generative AI use in educational settings

There are limitations that affect the validity of generative AI outputs, such as being trained on biased data and the generation of fake results, among others [7]. As indicated above, lesson plans generated by ChatGPT were reasonably defined and accurately reflected the desired pedagogical approaches but still needed teacher adaptation [8, 9]. In addition, the content generated by ChatGPT was “generally skewed towards content that reflects Western perspectives and people” [9, p. 450]. The outputs generated by AI rely on the data fed into the language model and so teachers may need to counteract bias by adapting lessons to be more inclusive.

An additional concern when it comes to using generative AI is that generative AI models have produced fake information [3, 5, 7, 10]. In one experiment highlighted by the Massachusetts School Library Association, ChatGPT identified a book as an article, listed a book with an incorrect author, and when prompted to use the MLA citation style, presented references in a fictitious style instead [11]. Cooper was concerned with the lack of supporting evidence for the various outputs and wondered about the risk that ChatGPT becomes situated as an “ultimate epistemic authority” [9, p. 449]. The risk of trusting in generative AI to provide truthful responses is that people that may not always be accurate or appropriate.

This study sought to build on the existing literature related to using generative AI to support lesson planning through the use of document analysis, in which the authors examined outputs by ChatGPT without involving any research participants. Document analysis is a systematic procedure for reviewing or evaluating documents—both printed and electronic [12, p. 27]. In determining the potential opportunities and risks associated with using generative AI for lesson planning, the following research questions guided our investigation of the efficiency and usefulness of using ChatGPT to design lesson plans.

### Research questions

1. Does generative AI facilitate the efficient generation of lesson plans?

#### Rationale 1

Although artificial intelligence has been around for over seven decades [13], its use in education, such as lesson planning, is a relatively new phenomenon. Researching the efficiency of ChatGPT-generated lesson plans will shed light on the potential value and implications of using generative AI in education.

2. To what extent did the first prompt in ChatGPT provide a lesson plan that aligned with the Massachusetts curriculum framework standard?

#### Rationale 2

Curriculum frameworks provide educators with guidance for implementing the content standards. Teachers are responsible for planning and delivering lessons, as well as assessing students’ learning [14].

3. What were the limitations or inaccuracies of lesson plans generated by ChatGPT?

#### Rationale 3

Lesson planning is essential for effective teaching. The primary goal of lesson planning is to create meaningful and purposeful learning experiences for students [15]. With students’ learning outcomes on the line, educators cannot afford to get it wrong. Recent research on the use of generative AI, such as ChatGPT, in creating lesson plans can provide teachers with specific directions and suggestions of readings for lesson plans [8]. Additionally, researchers have cautioned educators to critically evaluate the limitations and potential biases [8].

### Research design

The use of ChatGPT in curriculum development and lesson design is a relatively new concept to educators [4]. We employed an exploratory case study design [16] to understand the potential impact of teachers’ and teacher educators’ use of ChatGPT to develop lesson plans. In general, using case studies as a methodology is beneficial to researchers as they seek to answer research questions that ask what, why, and how [17]. We explored the capacity of ChatGPT 3.5 to create a lesson plan for first grade students in Massachusetts. Our experiment examined what ChatGPT could produce, why teachers might choose to use the tool, and how they might effectively use it.

### Strengths of case study approach

An exploratory case study explores situations where the intervention being evaluated has no clear or single outcome [17, 18]. A case study can provide researchers with a complete picture of a situation, phenomenon, or event [19] and is used in both qualitative and quantitative research [20]. Case study research provides opportunities to examine real-life situations closely [21] and study individual cases in-depth [22], which could lead to the creation of hypotheses [19], which in turn could lead to advancing knowledge of a field [23]. Jacobsen also states that the internal validity is very high, which makes these studies very valuable [19, p. 5]. Others have stated that case studies are well suited to asking how and why questions [17, 24].

### Limitations of case study approach

Critics have questioned the reliability and validity of case studies in research [21]. Case study research methods lack well-defined protocols [25] and create problems for researchers related to investigating causality and general conditions [22]. Others concluded that the findings and recommendations generated from case studies could neither be confirmed nor denied in terms of utility and veracity [26]. Flyvbjerg identified the following five disadvantages of case study research [21, p. 9]:

- Theoretical knowledge is more valuable than practical knowledge, and case studies can only provide the latter.
- One cannot generalize on the basis of an individual case; therefore, the case study cannot contribute to scientific development.
- The case study is most useful for generating hypotheses; that is, in the first stage of a total research process, while other methods are more suitable for hypotheses testing and theory building.
- The case study contains a bias toward verification, that is, a tendency to confirm the researcher’s preconceived notions.
- It is often difficult to summarize and develop general propositions and theories on the basis of specific case studies.

Recognizing the limitations of the case study approach, we determined that treating our interaction with ChatGPT as a case study facilitated documenting and analyzing our exploration of the tool. In addition, we respectfully disagree with Flyvbjerg’s assertion that a case study is unsuited to hypothesis testing and theory building. Our prompting of ChatGPT tested our theories about the efficiency and accuracy of ChatGPT to create lesson plans, which were based on previous exploratory case study designs [8, 9].

## Method

### Data collection

We set out to determine the effectiveness of using ChatGPT to create learning opportunities that allow students to engage in the constructivist way of learning rather than an overreliance on the teacher [27, 28]. As a result, we decided on the 5E Model of Instruction. This instruction model has been influential in science teaching and learning nationally and internationally [29]. In this investigation, we wanted to learn about the effectiveness of using ChatGPT in helping pre-service teachers create engaging lessons for early childhood students. We also wanted to know the extent to which the suggested lesson generated by ChatGPT was aligned to prompt input and to identify any potential limitations in using ChatGPT to create lesson plans.

We used the ChatGPT output as our qualitative data. To collect this data, we first prompted ChatGPT with the questions we devised in the order outlined below. The first question sought to replicate Cooper’s investigation of ChatGPT’s capacity to accurately define the 5E model [9]. Question 2 was the primary prompt to generate the first iteration of the lesson plan. Questions 3–9 resulted from our attempt to refine the lesson and ensure it was robust enough to encourage students to construct their knowledge of the differences and similarities between plants and animals through guided inquiry and hands-on experience.

#### Data analysis

To make meaning, gain understanding, and develop knowledge of the data (lesson plan) generated from ChatGPT, a document analysis [30] was done on the lesson plan generated. Bowen defined document analysis as a systematic procedure for reviewing or evaluating documents that are either printed or electronic [30]. In the case of our study, all documents received from ChatGPT were electronic. To reach a consensus, both authors independently read and re-read the data. We then discussed our interpretations and came up with additional prompts that we input in ChatGPT to refine the lesson plan to better reflect the desired learning outcome.

## ChatGPT and lesson plan iterations

**Fig 1:**
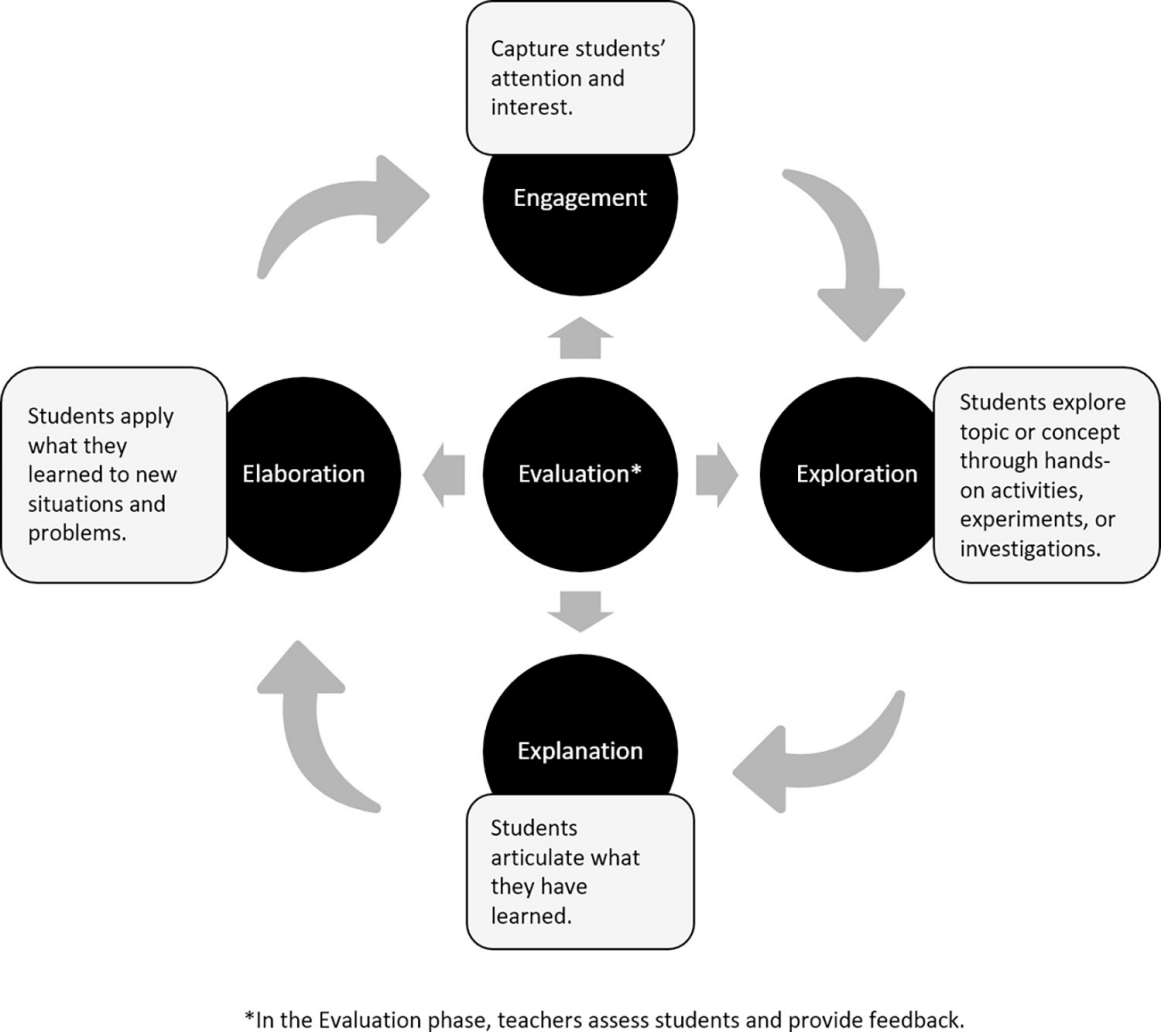
Definition of 5E model, adapted from ChatGPT output.

The first prompt (see Table 1, question 1) asked ChatGPT to provide a standard overview of the five phases of the 5E Model (Engagement, Exploration, Explanation, Elaboration, and Evaluation). In summary, Chat GPT stated that the 5E model is designed to be a cyclical process, with each stage building upon the previous one. The 5E model encourages active participation, critical thinking, and a deeper understanding of the subject matter. ChatGPT stated that the model is flexible and can be adapted to suit different educational contexts and grade levels, making it a valuable tool for science educators and teachers in various subjects. ChatGPT also included definitions for each of the five phases. Fig 1 presents excerpts of the ChatGPT definitions as a process diagram.

**Table 1:** List of prompts to ChatGPT.

Order	Prompt
1	What is the 5Es model of instruction?
2	You are an elementary school teacher teaching a science unit on heredity: inheritance and variation of traits. Create a lesson plan to teach children in Grade 1 to use information from observations (first-hand and from media) to identify similarities and differences among individual plants or animals of the same kind. The lesson plan should be structured according to the 5E Model. The lesson plan should align with the Massachusetts Department of Elementary and Secondary Education Science/Technology curriculum framework: 1-LS3-1 Use information from observations (first-hand and from media) to identify similarities and differences among individual plants or animals of the same kind [1-LS3-1.].
3	This lesson plan looks good. Can you revise it to make sure that the lesson plan includes opportunities for students to ask and answer questions about a text, opportunities to write, and opportunities to participate in discussion?
4	Can you suggest a text from Newsela for the short, age-appropriate text in the Engagement phase?
5	Ok. Can you suggest a text for the short, age-appropriate text in the Engagement phase?
6	Instead, please recommend a published book for the reading.
7	Instead of assigning homework, can we make the writing component a classroom activity?
8	Instead of the Engagement activity you created above, can you update the engage phase to ask students to participate in creating a KWL chart about plants and animals? During the engage phase, the class will complete the Know section. The lesson should also be updated so that the Want to Know and Learned sections of the KWL chart are completed during the Evaluation stage of the 5E lesson.
9	For the Elaboration phase, do you have access to a bank of photos that we could use?

### ChatGPT and the 5E model lesson iteration 1

Based on our assessment, ChatGPT provided an accurate overview of the 5E model, so we proceeded to create the lesson plan prompt (see Table 1, question 2). We wanted ChatGPT to create a lesson plan to teach first graders in the United States about heredity: inheritance and variations of traits. Specifically, we wanted to create a lesson plan that will provide opportunities for first grade students in the United States to use information from observations (first-hand and from media) to help them identify similarities and differences among individual plants or animals of the same kind [31, p. 35]. We also wanted ChatGPT to create a lesson plan that would define opportunities for students to engage in active learning and use critical thinking in age-appropriate ways as they develop their knowledge through guided inquiry and hands-on learning on the differences and similarities among organisms.

We carefully crafted the prompt before inputting it into ChatGPT since we knew specificity would provide a more nuanced response. The prompt provided us with a timed lesson plan with a title, grade level, curriculum framework standard, duration, materials, objectives, and timed elements of the 5E Model (see S1 Appendix). The lesson plan title (Exploring Similarities and Differences Among Plants and Animals) captured the prompt’s big idea. The curriculum framework required students to use information from observations (first-hand and from media) to identify similarities and differences among individual plants or animals of the same kind, so we considered how the lesson aligned with these expectations. We wanted to ensure the lesson was aligned so that any potential students exposed to it would master the grade level standard.

Although the material list provided by ChatGPT suggested that teachers could use pictures or illustrations of various plants and animals, an actual list of plants and animals was not provided. ChatGPT also suggested that teachers could use a live or plastic model of a common plant (e.g., a flower or tree) and an animal (e.g., a dog or cat). Therefore, teachers should read through the lesson output that ChatGPT provides to ensure they can use suggested items from the material list provided. These suggestions caused us to be very cautious of the efficiency of generative AI such as ChatGPT to generate lessons that are practical.

ChatGPT suggested a 10-minute timeframe for the engage portion of the lesson. In this lesson phase, ChatGPT indicated that the teacher would show the students pictures or illustrations of various plants and animals and ask the students if they have ever wondered why some animals look similar to their parents or why some plants have different colors and shapes. In this lesson phase, ChatGPT also indicated that the teacher should discuss with the students their prior knowledge and curiosity about how plants and animals are similar and different from one another. Although a veteran teacher might be easily able to engage students in discussion that taps into their prior knowledge and curiosity about how plants and animals are similar and different from one another, pre-service and new teachers might be less prepared to create opportunities for students to access prior knowledge [32]. Pre-service teachers might benefit from guiding questions to help students activate their prior knowledge. An additional risk is that pre-service teachers might be more likely to try using generative AI; Kelly and colleagues determined that college students under 25 years of age were more confident to use generative AI than students over 40 years old [33].

ChatGPT suggested a 15-minute timeframe for the exploration section of the lesson. In this phase, the teacher would introduce the live or plastic models of a common plant and animal to the students and encourage them to observe and discuss what they see. After this exercise, the teacher would divide the students into small groups and provide them with pictures or illustrations of different plants and animals. In their groups, the students would then be required to make observations and discuss the similarities and differences among the individual plants and animals. The lesson plan did not include a prompt for the teacher to provide guidance on how to structure the observation process.

ChatGPT suggested a 10-minute timeframe for the exploration section of the lesson. In this phase, ChatGPT suggested that the teacher bring the class back together and have each group share their observations. ChatGPT encouraged the teacher to create a class chart on the whiteboard or chart paper to record the similarities and differences that the students discovered. To further enhance the students’ understanding of differences and similarities between plants and animals, ChatGPT suggested that teachers explain to the students that just as people have similarities and differences, plants and animals do, too. Also, ChatGPT stipulated that the teacher discuss similarities and differences between parents and their offspring. ChatGPT did not suggest guiding discussion questions, but we believe the lesson suggestions in the explanation phase will help students understand the differences and similarities among plants and animals.

ChatGPT suggested a 15-minute timeframe for the elaboration section of the lesson. In the elaboration phase, teachers could explore heredity and variation further by showing students photos or videos of different plant and animal offspring with their parents (e.g., a puppy with its dog parent, a baby bird with its bird parent). After showing these to students, the teacher could encourage them to discuss how the offspring are similar to their parents and what traits they inherit. Additionally, the teacher could have students work in pairs to draw and label pictures of a plant or animal and its offspring, highlighting their similarities and differences. The teacher might need to teach students the meaning of words such as heredity, variation, traits, and offspring. ChatGPT’s lesson plan did not suggest that teachers consider whether and how to teach these terms to students.

A 10-minute timeframe was suggested for the evaluation phase. In this phase, ChatGPT suggested that teachers conclude the lesson by reviewing the key concepts of heredity and variation of traits and ask students to share what they learned. The teacher should then give each student a science journal or worksheet and ask them to draw and write about a plant or animal they find interesting, highlighting its traits and any similarities and differences they observe. As a homework assignment, ChatGPT suggested that teachers encourage students to observe plants and animals in their environment and record their observations in their science journals. It is interesting to note that ChatGPT recommended the teacher assign homework to first-grade students. We judged that this could be a realistic assignment at a first-grade level, but we questioned whether first-grade students would normally be assigned homework of this nature.

At the end of the lesson, ChatGPT provided a summary, advocating that the teacher assess students’ understanding through class participation, discussion, and journals or worksheets. In addition, ChatGPT claimed that the lesson plan aligned with the Massachusetts curriculum framework. Finally, ChatGPT provided a form of disclaimer acknowledging that the teacher should adapt the lesson plan to meet the specific grade level and students’ needs and abilities. See S1 Appendix for the complete output.

### ChatGPT and the 5E model iteration 2

To test ChatGPT’s capacity to iterate and modify its output, we asked follow-up questions to refine the lesson. The Massachusetts curriculum framework includes the science and engineering practices as guiding principles for effective science and technology/engineering education in the Commonwealth of Massachusetts. Therefore, we asked ChatGPT to revise the lesson plan to include opportunities for students to ask and answer questions about a text, opportunities to write, and opportunities to participate in discussion (see Table 1, Question 3). In the output that followed, ChatGPT left the explanation and evaluation phases of the lesson unchanged, but it modified the material list, as well as the engage, elaboration, and exploration phases.

In lesson iteration 2 (see S2 Appendix), our prompt yielded an additional item in the lesson’s material list, a short, age-appropriate text or story related to heredity and traits. The engage section of the lesson now asked the teacher to read a short-age-appropriate text or story related to heredity, traits, and the similarities and differences among plants and animals. The teacher was also asked to encourage their students to listen carefully and ask questions about the text as it was read to them. In this phase, it was also suggested that teachers initiate a class discussion by asking students what questions they have about heredity and why some plants and animals look similar or different and discuss what they see.

ChatGPT made a slight adjustment to the exploration section of the lesson, directing students to write down questions about animals and plants before discussing their similarities and differences. The main change in the elaboration section was for the students to write down questions as they arise. However, ChatGPT did not provide directions on whether or not the teacher should respond to any questions that might arise. We noted that failing to prompt teachers to address students’ questions introduces a risk; the teacher might miss an opportunity to ensure that concepts are clear to students and subsequently achieve lesson closure. However, this revised lesson plan continued to align with the Massachusetts curriculum framework (1-LS3-1) while incorporating opportunities for questioning, writing, and discussion.

### Reading recommended by ChatGPT

We wanted the students to engage in reading, so we prompted ChatGPT to adapt the lesson plan to provide opportunities for students to read texts (see Table 1, Prompts 4–6). First, we asked if ChatGPT could suggest a text from Newsela, an online literacy platform that features a wide range of current articles that are commonly used by K-12 teachers. However, ChatGPT replied that it cannot access external sites like Newsela. We then asked ChatGPT to recommend an age-appropriate text for use in the engagement phase. Rather than recommending a published text, ChatGPT provided what appeared to be an original work of its creation, *Why Do Some Kittens Look Like Their Cat Parents*? No author was provided, and we were unable to confirm if the output was a published work or an original creation by ChatGPT. ChatGPT did provide guiding questions for discussion. Earlier iterations of the lesson had not included such questions, so we were excited to see this development. However, after searching for the title, we were not sure if the story was an actual published work, which led us to prompt ChatGPT to recommend a published text. ChatGPT recommended, *Why Do Tigers Have Stripes*? *And Other Questions About Evolution* by Mary Kay Carson.

According to ChatGPT, “This book is part of a series that explores questions about animals, plants, and evolution.” We searched Amazon books and university library databases. We contacted a local high school librarian to aid us in our search. We were able to find the author, Mary Kay Carson, but we failed to locate the ChatGPT-recommended text. Therefore, we want to caution teachers of the potential limitations or inaccuracies of lesson plans generated by ChatGPT. Teachers who plan to use ChatGPT to create lesson plans should always review suggested activities and resources.

## Findings by research question

### Research question 1

For the first research question on whether generative AI facilitated efficient generation of lesson plans, we produced and substantively adapted a lesson within 30 minutes. We prompted the creation of the first lesson plan (Prompt 2 listed in Table 1) and then generated five follow-up questions to clarify and revise the lesson (Prompts 3–7 in Table 1). Following each output from ChatGPT, we reviewed the content of the output and discussed our reactions. ChatGPT produced variations of the lesson plan within seconds of each follow-up prompt. Therefore, ChatGPT facilitated a quick process for lesson planning.

### Research question 2

For the second research question on the extent to which the first prompt in ChatGPT provided a lesson plan that aligned with the curriculum framework standard, we compared the lesson plan generated by ChatGPT with the Massachusetts Department of Elementary and Secondary Education Science/Technology curriculum framework: 1-LS3-1 Use information from observations (first-hand and from media). The lesson output asked students to identify similarities and differences among individual plants or animals of the same kind, which aligned with the curriculum framework requirement. We followed up with a question asking ChatGPT to ensure that the lesson plan included opportunities for students to ask and answer questions about a text, opportunities to write, and opportunities to participate in discussion. These are important tenets of the Science and Engineering Practices [34]. For example, students are expected to ask questions and define scientific problems, engage in arguments from evidence, and obtain, evaluate, and communicate information. Therefore, the lesson plan aligned appropriately with the curriculum framework.

### Research question 3

For the third research question on potential limitations or inaccuracies of lesson plans generated by ChatGPT, we found that the lesson plans generated by ChatGPT were generally accurate and helpful, but included problematic lesson components, missing details, and one inaccuracy. A problematic lesson component we identified was in the exploration phase of iteration 1. The lesson plan asked students to make observations about the similarities and differences among individual plants and animals. Although this task could potentially be an excellent opportunity to help students develop their observation skills, a risk is that people often have difficulties differentiating between inference and observation, such as a tendency to state assumptions as observation [35]. Therefore, it is concerning for ChatGPT to suggest an activity that requires 1st graders to make observations and discuss similarities and differences but not provide guidance on how to conduct observations. Experienced teachers and education faculty could help pre-service and new teachers avoid this pitfall.

There were additional problematic components in the lesson plan generated by ChatGPT. One concern arose from the elaboration phase, which involved student exploration of heredity and variation without including time to discuss the meaning of these concepts in detail.

**Fig 2:**
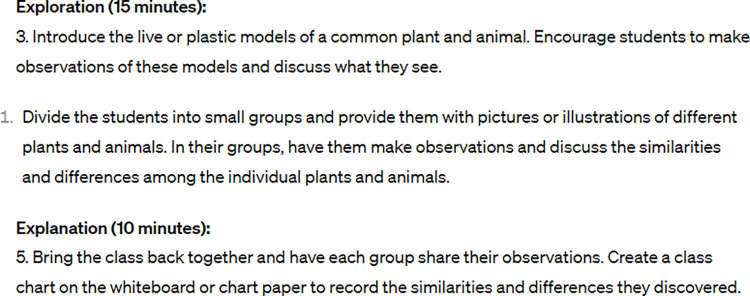
Excerpt from ChatGPT output: Introduce live plant or animal.

A third potentially problematic component was that ChatGPT suggested that teachers could potentially use live animals, among other options, for making comparisons (Fig 2). However, many schools across the United States might not allow pets, such as dogs or cats, in the classroom out of precaution for students’ health and safety, and the safety practices and legal requirements for proper treatment [36].

The lesson plan generated by ChatGPT was missing certain details and ended with a recommendation to, “Remember to adapt the lesson plan to the specific needs and abilities of the students, and feel free to incorporate any additional resources or media that may enhance the learning experience.” There were two explicit examples of missing details. The ChatGPT-generated lesson recommended the use of imagery to compare different plants and animals, but the teacher would need to locate and select the pictures to be used. Second, the teacher was asked to discuss similarities and differences between parents and offspring, but the lesson plan did not include discussion prompts that the teacher could adopt.

There were also more subtle examples of missing details that could easily be missed by an inexperienced teacher. For example, students were prompted to observe similarities and differences, but without guidance on how to structure the observation process. In addition, the teacher was asked to have conversations with the students about concepts of heredity, variation, and traits, but the lesson did not include a clear moment for when and how these terms would be appropriately defined for the first-grade students.

**Fig 3:**
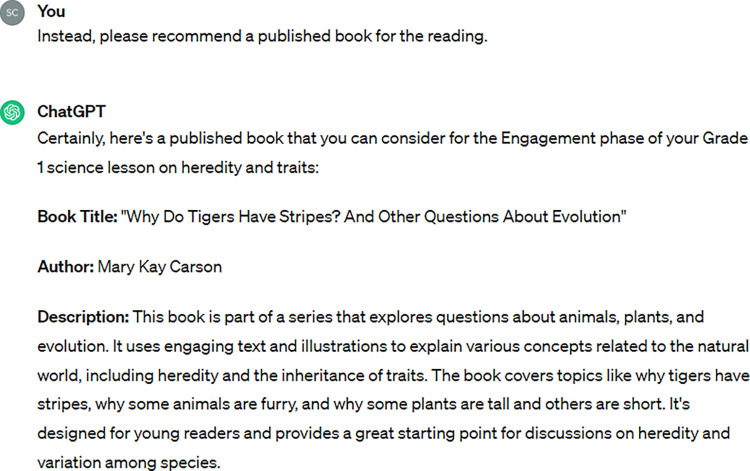
ChatGPT hallucinated book recommendation.

A final concern about the lesson plan was an inaccuracy. During the engagement phase, ChatGPT suggested a book by Mary Kay Carson, *Why Do Tigers Have Stripes*? *And Other Questions about Evolution* to learn about heredity and traits (Fig 3). Although the author exists and has written similar books, this book was hallucinated by ChatGPT. The case of invented or fake information provided by generative AI tools has been documented and commonly referred to as “hallucinations” [2, 5, 7]. In this instance, ChatGPT invented a fake book by a real author.

## Implications for practice

If generative AI tools can be useful supports for lesson planning, then teachers need to have a skeptical disposition regarding AI outputs. Therefore, education department faculty in college have an important role in teaching pre-service teachers why, how, and when to use generative AI in the classroom. In terms of helping pre-service teachers to understand the *why*, Chan and Zhou found a correlation between individuals’ perceived value of generative AI and their intention to use it [37]. Encouraging future teachers to use generative AI might not be a sufficient intervention; some individuals need to understand the value of the tool before they will adopt it. Therefore, education faculty can help pre-service teachers understand why and how generative AI can be valuable, such as to help teachers with efficiently designing lessons.

An additional potential role for education faculty is to ensure a rigorous examination of generative AI outputs. Faculty can scaffold pre-service teachers’ approach to *how* and *when* it is appropriate to employ AI tools. According to Kelly and colleagues, younger college students were more likely to feel confident that they can use generative AI, but mainly learned about it from social media [33]. Faculty can scaffold students’ ability to critically examine generative AI outputs. For example, to address Cooper’s concern with the lack of supporting evidence in outputs, faculty can encourage pre-service teachers to locate authoritative and appropriate sources to support the information produced by generative AI [9].

Our assessment of the problematic lesson components, missing details, and inaccuracies, as well as the disclaimer provided by ChatGPT, showcase the need for teachers to proceed with caution if and when they use AI to create lessons. Teachers must read over a ChatGPT provided lesson and make changes to align it to the standard they require their students to master. Teachers who decide to use generative AI to create a lesson plan should carefully think through the prompts they input to help them achieve high quality lesson plans, aligned to curriculum standards, and that meet the needs of their students. Once lesson plans are generated, teachers then have to critically examine and potentially modify the outputs to ensure that the lesson is accurate and aligns with the local context.

## Future research directions

We used an exploratory case study design to facilitate researcher testing of generative AI. ChatGPT responded to the prompts we devised and we performed document analysis on the outputs. Recent research on using generative AI for lesson planning has used a similar approach and achieved comparable findings that ChatGPT can produce a good first draft of a lesson plan, but a critical revision process is also necessary [8, 9]. The consistency and reproducibility of generative AI responses remain a concern; Franc and colleagues indicated that inaccuracy and inconsistency made ChatGPT insufficient for emergency medical triage [38]. Future research could continue to probe generative AI tools to take stock of their capabilities and accuracy for use in designing instruction, including each tool’s capacity to reflect contextual elements, such as grade level and school culture.

An additional direction for research would be to study the authentic use of generative AI by in-service and pre-service teachers. One approach could focus on understanding the users’ perspective, how they value generative AI tools and how they put them to use. Another approach would be to evaluate interventions to scaffold pre-service undergraduate students in critically evaluating generative AI outputs. Researchers and education faculty could partner to train students to recognize generative AI responses to be a starting point rather than an outcome.

## Conclusion

The use of generative AI, such as ChatGPT, is poised to have lasting effects on how teachers plan their daily lessons. The present study found that ChatGPT efficiently produced a lesson plan that accurately used the 5E model. However, using generative AI should not replace the teacher’s expertise related to effective pedagogical practices and the contextual factors required to help students achieve academic success. In this investigation, we found that teachers would need to strengthen the AI-generated lesson plan to replace problematic components, fix inaccuracies, and fill in missing details. For example, the teacher could consider strategies for scaffolding student mastery of the key concepts in the lesson, such as by defining the concepts, using lesson discussion prompts, and by selecting the most appropriate materials for the lesson.

The lesson plan generated by ChatGPT provided a helpful starting point for teachers to plan learning opportunities for their students. However, certain elements were left undefined or were arguably inappropriate for first-grade classrooms. Therefore, the teacher must critically analyze the output to assess limitations, ask follow-up questions, and make modifications. It is critically important that teachers refine their prompts to ChatGPT to get outputs that align well with the curriculum standard to be addressed by the lesson. Our experiment with ChatGPT and lesson planning generated additional evidence for the value proposition and the caution needed to successfully use generative AI. ChatGPT proposed that we use a text that does not exist. Educators should avoid solely relying on AI to generate lesson plans in light of the potential limitations. These limitations could impact the lesson plan’s potential to help students achieve academic success.

## Supporting information

S1 Appendix (DOCX)

S2 Appendix (DOCX)
