# Supplementary material for: Opportunities and risks involved in using ChatGPT to create first grade science lesson plans
Source: PLoS One. 2024 Jun 17;19(6):e0305337. doi: 10.1371/journal.pone.0305337 (PMC11182495; doi:10.1371/journal.pone.0305337)
Supplement: S1 Appendix — (DOCX) [file pone.0305337.s001.docx]

Appendix A

**Prompt:** You are an elementary school teacher teaching a science unit on heredity: inheritance and variation of traits. Create a lesson plan to teach children in Grade 1 to use information from observations (first-hand and from media) to identify similarities and differences among individual plants or animals of the same kind. The lesson plan should be structured according to the 5E Model. The lesson plan should align with the Massachusetts Department of Elementary and Secondary Education Science/Technology curriculum framework: 1-LS3-1 Use information from observations (first-hand and from media) to identify similarities and differences among individual plants or animals of the same kind.

**ChatGPT:**

Certainly! Here's a sample lesson plan following the 5E model to teach Grade 1 students about heredity, inheritance, and variation of traits. This lesson aligns with the Massachusetts Department of Elementary and Secondary Education Science/Technology curriculum framework (1-LS3-1).

**Title:** Exploring Similarities and Differences Among Plants and Animals

**Grade Level:** 1

**Duration:** Approximately 60 minutes

**Materials:**

- Pictures or illustrations of various plants and animals
- Live or plastic model of a common plant (e.g., a flower or tree) and an animal (e.g., a dog or cat)
- Whiteboard and markers
- Chart paper and markers
- Science journals or worksheets for each student
- Computer or tablet for displaying media (optional)
- Photos or videos of different plants and animals (optional)

**5E Lesson Plan:**

**Engagement** (10 minutes): Begin the lesson by showing students pictures or illustrations of various plants and animals. Ask the students, "Have you ever wondered why some animals look similar to their parents? Or why some plants have different colors and shapes?"

Discuss with the students their prior knowledge and curiosity about how plants and animals are similar and different from one another.

**Exploration** (15 minutes): 3. Introduce the live or plastic models of a common plant and animal. Encourage students to make observations of these models and discuss what they see.

Divide the students into small groups and provide them with pictures or illustrations of different plants and animals. In their groups, have them make observations and discuss the similarities and differences among the individual plants and animals.

**Explanation** (10 minutes): 5. Bring the class back together and have each group share their observations. Create a class chart on the whiteboard or chart paper to record the similarities and differences they discovered.

Explain to the students that just like people have similarities and differences, plants and animals do too. Discuss that some of these similarities and differences are passed down from parents to their offspring.

**Elaboration** (15 minutes): 7. To explore heredity and variation further, you can show photos or videos of different plant and animal offspring with their parents (e.g., a puppy with its dog parent, a baby bird with its bird parent).

Encourage the students to discuss how the offspring are similar to their parents and what traits they inherit.

Have students work in pairs to draw and label pictures of a plant or animal and its offspring, highlighting their similarities and differences.

**Evaluation** (10 minutes): 10. Conclude the lesson by reviewing the key concepts of heredity and variation of traits. Ask students to share what they've learned.

Provide each student with a science journal or worksheet. Ask them to draw and write about a plant or animal they find interesting, highlighting its traits and any similarities and differences they observe.

**Homework:** Encourage students to observe plants and animals in their environment and record their observations in their science journals.

**Assessment:** Assess students' understanding through their class participation, group discussions, and the content of their science journals or worksheets. This lesson plan aligns with the Massachusetts curriculum framework (1-LS3-1) by focusing on using information from observations to identify similarities and differences among individual plants and animals of the same kind.

Remember to adapt the lesson plan to the specific needs and abilities of your Grade 1 students, and feel free to incorporate any additional resources or media that may enhance the learning experience.
