# Supplementary material for: Opportunities and risks involved in using ChatGPT to create first grade science lesson plans
Source: PLoS One. 2024 Jun 17;19(6):e0305337. doi: 10.1371/journal.pone.0305337 (PMC11182495; doi:10.1371/journal.pone.0305337)
Supplement: S2 Appendix — (DOCX) [file pone.0305337.s002.docx]

Appendix B

**Prompt:** This lesson plan looks good. Can you revise it to make sure that the lesson plan includes opportunities for students to ask and answer questions about a text, opportunities to write, and opportunities to participate in discussion?

**ChatGPT:**

Certainly! I've revised the lesson plan to ensure that it includes opportunities for students to ask and answer questions about a text, opportunities to write, and opportunities to participate in discussion.

**Title:** Exploring Similarities and Differences Among Plants and Animals

**Grade Level:** 1

**Duration:** Approximately 60 minutes

**Materials:**

- Pictures or illustrations of various plants and animals
- Live or plastic model of a common plant (e.g., a flower or tree) and an animal (e.g., a dog or cat)
- Whiteboard and markers
- Chart paper and markers
- Science journals or worksheets for each student
- Computer or tablet for displaying media (optional)
- Photos or videos of different plants and animals (optional)
- A short, age-appropriate text or story related to heredity and traits

**5E Lesson Plan:**

**Engagement** (10 minutes): Begin the lesson by reading a short, age-appropriate text or story related to heredity, traits, and the similarities and differences among plants and animals. Encourage students to listen carefully and ask questions about the text as you read.

After reading, initiate a class discussion by asking students what questions they have about heredity and why some plants and animals look similar or different from each other.

**Exploration** (15 minutes): 3. Introduce the live or plastic models of a common plant and animal. Encourage students to make observations of these models and discuss what they see.

Divide the students into small groups and provide them with pictures or illustrations of different plants and animals. In their groups, have them make observations, write down questions they have about the animals and plants, and discuss the similarities and differences among them.

**Explanation** (10 minutes): 5. Bring the class back together and have each group share their observations and questions about the animals and plants.

Create a class chart on the whiteboard or chart paper to record the similarities and differences they discovered.

Explain to the students that just like people have similarities and differences, plants and animals do too. Discuss that some of these similarities and differences are passed down from parents to their offspring.

**Elaboration** (15 minutes): 8. To explore heredity and variation further, you can show photos or videos of different plant and animal offspring with their parents (e.g., a puppy with its dog parent, a baby bird with its bird parent).

Encourage the students to discuss how the offspring are similar to their parents and what traits they inherit. Write down students' questions as they arise.

Have students work in pairs to draw and label pictures of a plant or animal and its offspring, highlighting their similarities and differences. They should also write down any questions they have about these traits.

**Evaluation** (10 minutes): 11. Conclude the lesson by reviewing the key concepts of heredity and variation of traits. Ask students to share what they've learned and the questions they've generated.

Provide each student with a science journal or worksheet. Ask them to draw and write about a plant or animal they find interesting, highlighting its traits, and questions they have.

**Homework:** Encourage students to ask their families about heredity and traits and write down any information they learn.

**Assessment:** Assess students' understanding through their active participation in discussions, their ability to ask questions about the text and the content they've explored, as well as the content of their science journals or worksheets. This revised lesson plan continues to align with the Massachusetts curriculum framework (1-LS3-1) while incorporating opportunities for questioning, writing, and discussion
